# Individual differences in toddlers’ social understanding and prosocial behavior: disposition or socialization?

**DOI:** 10.3389/fpsyg.2015.00600

**Published:** 2015-05-11

**Authors:** Rebekkah L. Gross, Jesse Drummond, Emma Satlof-Bedrick, Whitney E. Waugh, Margarita Svetlova, Celia A. Brownell

**Affiliations:** ^1^Early Social Development Lab, Department of Psychology, University of Pittsburgh, Pittsburgh, PAUSA; ^2^Department of Developmental and Comparative Psychology, Max Planck Institute for Evolutionary Anthropology, LeipzigGermany

**Keywords:** prosocial behavior, social understanding, temperament, parent socialization, individual differences

## Abstract

We examined how individual differences in social understanding contribute to variability in early-appearing prosocial behavior. Moreover, potential sources of variability in social understanding were explored and examined as additional possible predictors of prosocial behavior. Using a multi-method approach with both observed and parent-report measures, 325 children aged 18–30 months were administered measures of social understanding (e.g., use of emotion words; self-understanding), prosocial behavior (in separate tasks measuring instrumental helping, empathic helping, and sharing, as well as parent-reported prosociality at home), temperament (fearfulness, shyness, and social fear), and parental socialization of prosocial behavior in the family. Individual differences in social understanding predicted variability in empathic helping and parent-reported prosociality, but not instrumental helping or sharing. Parental socialization of prosocial behavior was positively associated with toddlers’ social understanding, prosocial behavior at home, and instrumental helping in the lab, and negatively associated with sharing (possibly reflecting parents’ increased efforts to encourage children who were less likely to share). Further, socialization moderated the association between social understanding and prosocial behavior, such that social understanding was less predictive of prosocial behavior among children whose parents took a more active role in socializing their prosociality. None of the dimensions of temperament was associated with either social understanding or prosocial behavior. Parental socialization of prosocial behavior is thus an important source of variability in children’s early prosociality, acting in concert with early differences in social understanding, with different patterns of influence for different subtypes of prosocial behavior.

## Introduction

Remarkably, while still learning how to speak, toddlers in their second and third years of life are attentive to their own and others’ internal states and begin engaging in prosocial behaviors such as helping and sharing. Yet, from the outset young children exhibit wide variability in the complexity and frequency of prosocial action (e.g., Brownell et al., 2006, 2013a; Warneken and Tomasello, 2006; Warneken et al., 2006; Svetlova et al., 2010; Dunfield et al., 2011). To engage in prosocial behavior, one must be able to recognize another’s goal, desire, or internal state, and respond to another’s needs by intervening to alter their subjective state. Thus, even in its earliest manifestations, prosocial behavior such as helping and sharing depends on and reflects social perception and social understanding (Vaish and Warneken, 2012; Brownell et al., 2013b).

Social understanding – the ability to infer others’ internal states such as goals, feelings, and desires – has its origins in the first year of life and gives rise to a variety of other-oriented behaviors at the beginning of the second year (Tomasello et al., 2005). Because social cognition is necessary for prosocial behavior, we might reasonably expect that individual differences in social cognition would relate to individual differences in prosocial behavior. And, indeed, this is known to be the case for older children in whom both social cognition and prosocial behavior are well-developed (Eisenberg et al., 2015). However, there are few studies that examine how variability in very early social understanding relates to emerging prosocial behavior. Thus, their association is not yet clear during the period when prosocial behavior first emerges, and when both systems are undergoing rapid and dramatic change. The current study uses multiple measures of social understanding and prosocial behavior to examine this association in one- and 2-years-old children. The study also includes measures of temperament and parent socialization to explore possible sources of individual differences in toddlers’ social understanding and prosocial behavior.

### Relations between Social Understanding and Prosocial Behavior in Toddlers

While prosocial behavior, such as handing a blanket to someone who is cold, may seem simple on the surface, such acts require complex understanding and action. The child must recognize that someone else has a problem, which may include understanding emotional facial expressions and their relations to others’ internal feelings; understanding that the distressed party is a separate entity with unique desires and goals, while also regulating one’s own desires and emotional responses; and understanding the specific type of assistance required and how to intervene, and adapting to any obstacles along the way. A small body of research has demonstrated connections between social understanding and some aspects of prosocial behavior in toddlers. For example, emotion and self-other understanding in 12- to 24-months-old were related to empathic responsiveness to a peer’s distress (Nichols et al., 2009). Fifteen-months-old infants who were more sensitive to unfair outcomes were also found to be more willing to share their preferred toy with an adult (Sommerville et al., 2013). Other studies have found associations between empathic concern, self- and other-understanding, and emotion understanding in young children (Bischof-Köhler, 1991; Zahn-Waxler et al., 1992; Ensor and Hughes, 2005; Garner et al., 2008).

Prosocial behavior may be best conceptualized as a multidimensional construct, at least in the early years (Eisenberg and Spinrad, 2014). Indeed, distinct prosocial behaviors are often uncorrelated in toddlers (Dunfield et al., 2011) and have different neural signatures in the second and third years of life (Paulus et al., 2013). Thus, some prosocial behaviors may rely more heavily on social understanding, such as those that involve inferring an internal state (e.g., empathic helping), in contrast to others that are more strictly goal-oriented (e.g., instrumental helping). Hence, the current study builds on existing research by including measures of several distinct types of prosocial behavior to explore whether social understanding plays a different role for different types of prosocial action.

### Individual Differences in Early Social Understanding and Prosocial Behavior

Questions about relations between variability in social understanding and variability in prosocial behavior are fundamentally individual difference questions: to what extent do within-age differences in social understanding account for differences in prosocial behavior? Yet these are also normative developmental phenomena – all typically developing children achieve the basic abilities to help, share, comfort, and cooperate with others, and to represent and act on their own and others’ internal subjective states. One source of variability in such normative developments is differences in rates of development. That is, at any given age, some children may be more advanced than others in social cognition and/or social behavior. One potential contributor to such within-age differences in competence may be parents’ socialization of their children’s responses to others’ emotions and behavior (Brownell, 2013; Denham et al., 2015; Eisenberg et al., 2015). Parent socialization, considered broadly, comprises the myriad ways parents help their children to become members of the social group. Socialization is a function of parental beliefs, goals, and values; occurs within many different contexts including play as well as discipline; and includes behavior ranging from the subtle (e.g., praising process vs. outcome) and indirect (e.g., monitoring) to more explicit and didactic (e.g., rewarding; coaching; Eisenberg et al., 1998; Grusec and Hastings, 2007).

A second source of individual differences in social understanding and/or prosocial behavior may be dispositional differences in children’s attention to or interest in social and emotional information or social engagement. For example, research has suggested the existence of an “empathic disposition,” such that some young children are dispositionally more likely than others to empathize with others in distress, regardless of their early socialization experiences (Nichols et al., 2009). Other dispositional or temperamental differences may affect children’s attentiveness to others. These possibilities are considered more fully below.

### Parental Socialization

Parental socialization of everyday activities is a known contributor to both prosocial behavior and social understanding throughout childhood (Denham et al., 2015; Hastings et al., 2015). Previous research has revealed that parents utilize a variety of socialization strategies to encourage young children’s developing prosocial behaviors, including negotiation (Crockenberg and Litman, 1990), scaffolding (Hammond et al., 2012), and praise (Grusec, 1991) and that socialization approaches vary with the age of the toddler (Pettygrove et al., 2013; Dahl, 2015; Waugh et al., 2015). Research has also shown that the content and context of parent socialization are associated with young children’s prosocial behavior. Toddlers whose mothers scaffolded their everyday helping were more helpful toward an unfamiliar adult (Pettygrove et al., 2013; Hammond and Carpendale, 2015). Those whose parents engaged them in more emotion-related discourse during joint book reading, and who were particularly asked by their parents to attend to and reflect on others’ emotions, helped more quickly and more often on emotionally laden helping and sharing tasks with other adults (Garner et al., 2008; Brownell et al., 2013c; Drummond et al., 2014b). Thus, parents socialize early-appearing prosocial behavior in multiple ways, both by scaffolding very early instances of prosocial responding and by drawing their children’s attention to the mental and emotional states of those around them.

Parent socialization may similarly influence developing social cognition (Carpendale and Lewis, 2004). Parents’ self-reported beliefs about the importance of teaching children about emotions, as well as their self-reported attention to and encouragement of their young children’s emotions and their observed responses to children’s emotions were associated with emotion understanding longitudinally in 3- and 5-years-old (Denham et al., 1994; Denham and Kochanoff, 2002). A number of researchers have reported associations between parent–child mental state talk and preschool children’s understanding of emotions and other psychological states (e.g., Lagattuta and Wellman, 2002; Ruffman et al., 2002; Taumoepeau and Ruffman, 2006; LaBounty et al., 2008), particularly when the discourse occurs within socially connected interchanges (Ensor and Hughes, 2008). Among younger children, those with more sensitive, “mind-minded” parents who respond to their infants “as individuals with minds” tend to later exhibit more advanced mentalizing abilities such as false-belief understanding (Meins and Fernyhough, 1999). During the second year of life, as toddlers begin to label their own and others’ emotions, parents also begin to discuss emotions in causal terms (Bretherton et al., 1986). Thus, as with prosocial behavior, parents socialize early-developing social understanding in multiple ways, both direct and indirect.

Building on this empirical base, the current study examined potential associations between parents’ self-reported socialization practices and their toddler-aged children’s social understanding and prosocial behavior. In particular, we asked parents to report their everyday socialization practices that focused on scaffolding and encouragement of the child’s prosocial behavior as well as on the child’s attention to and discussion of emotions.

### Temperament

Several studies have demonstrated associations between specific dimensions of temperament and social understanding in young children. Using standard false-belief theory of mind (ToM) tasks and parent-reported temperament measures, Wellman et al. (2011) found that a shy-withdrawn temperament at age 3 predicted later ToM understanding at age 5. Shyness in children as young as 18 months has been found to be positively correlated with ToM understanding at 3 years of age (Mink et al., 2014). Similarly, children who tended to observe their peers rather than play actively with them, possibly denoting shyness or social cautiousness, were more advanced in ToM (Moore et al., 2011). Wellman et al. (2011) reasoned that a shy-observant, possibly more regulated and cautious, approach to social interaction might enhance children’s ability to take a reflective stance on others’ behavior, thereby contributing to a developing understanding of its causes in underlying mental states. While previous research provides insight into the relationship between temperament and social understanding during the preschool years, to our knowledge no research has tested the concurrent associations between a cautious, shy, possibly more fearful temperament and social understanding in infants; this is one of the aims of the current study.

Associations between temperamental fearfulness and prosocial responding have also been examined, with mixed results. Spinrad and Stifter (2006) reported that fearfulness assessed at 10 months of age predicted greater concern toward a distressed adult at 18 months of age. In contrast, van der Mark et al. (2002) found that fearfulness observed at 16 months of age was associated with reduced empathic concern at 22 months; and Liew et al. (2011) found no association between fearfulness at 18 months and concern for another’s distress at 30 months. Moreover, neither Spinrad and Stifter (2006) nor Liew et al. (2011) found any links between temperamental fear and actual prosocial behavior such as comforting or helping. Whether these inconsistent findings reflect variation in how temperament was measured, the particular dispositional constructs assessed (e.g., fearfulness vs. shyness), or different patterns in younger children than in older children is unknown. It thus remains an open question whether or how early temperament relates to early prosocial behavior. The current study adds to this literature by exploring concurrent associations between fearfulness, shyness, and social fearfulness and prosocial behavior in 18- to 30-months-old toddlers.

In sum, the first goal of the current study was to determine whether and how variability in toddlers’ social understanding predicted their prosocial behavior. We assessed social understanding using parent report of their children’s self-other differentiation and emotion-related vocabulary. We included multiple types of prosocial behavior, both as it occurs in the family environment as reported by parents and as observed in lab tasks of sharing and helping with other adults. We then examined two potential sources of individual variability in social understanding and prosocial behavior; specifically, parent socialization and specific dimensions of temperament known to predict social understanding in preschoolers and sometimes to predict prosocial behavior in toddlers.

## Materials and Methods

### Participants

Participants were drawn from five previously completed studies of early prosocial behavior. They included 135 18-months-olds (*M* = 18.32, SD = 0.681; girls = 65; boys = 70), 56 24-months-old (*M* = 23.36, SD = 1.45; girls = 27; boys = 29), and 134 30-months-old (*M* = 29.18, SD = 1.03; girls = 59; boys = 75). Families were recruited from a medium-sized city and surrounding suburbs and received a small book or toy for completing the study. All participants were healthy and typically developing. Eighty-two percent were Caucasian, 7% were biracial, 4% were African-American, 3% were Asian, 1% was Hispanic, and 3% did not disclose their race. No participants took part in more than one of the studies. University IRB approval was obtained prior to initiating each study, SRCD ethical guidelines were followed, and parents provided written consent for their own and their children’s participation.

### General Procedure

Procedures were similar for each study. After a brief warm-up play period in a separate room with an experimenter (E) and an assistant experimenter (AE), the child and a parent were escorted to a playroom where the study procedures were conducted. All sessions were video-recorded from behind a one-way mirror. During the study, the parent remained in the room with the child, filling out questionnaires; parents were asked not to instruct or encourage their children during the session, but otherwise to respond naturally to their children’s communications or social bids. Parents completed questionnaires rating children’s temperament, social understanding, prosocial behavior, and parental socialization practices (details below). Additionally, children participated in sharing and/or helping tasks with E in each study. Brief periods of free play with a standard set of toys occurred between prosocial tasks; in some cases, children completed additional procedures for a larger study between the prosocial tasks. Within each study, all tasks were administered within-subjects and counterbalanced for order.

The helping and sharing tasks followed a similar format. For each one, E needed an object or objects to which the child had access but which were out of E’s reach. The child could alleviate E’s need or desire by giving her or him one or more relevant objects. Two types of helping tasks were administered: instrumental helping tasks in which E dropped or misplaced an object that s/he needed to complete a goal-directed action; and empathic helping tasks in which E experienced a negative internal state such as being cold or sad that could be alleviated by a blanket or a favorite toy. In the sharing tasks the child had an abundance of objects (e.g., cars, zoo animals) and E had none. On each of the helping and sharing tasks E delivered a standard series of cues, which became progressively more detailed and specific about E’s need or desire and how it could be alleviated. No thanks or praise was provided when children helped or shared with E. Children were given a score of 1 if they helped or shared on any trial and a score of 0 if they did not help or share on any trial. This dichotomous measure of helping is commonly used when evaluating prosocial behavior in infants and toddlers (e.g., Warneken and Tomasello, 2006, 2007; Over and Carpenter, 2009; Dunfield et al., 2011; Sommerville et al., 2013).

Details for each study are provided below. Because some of the measures differed across studies (e.g., temperament; sharing), different subsamples of participants contributed to the analyses. **Table 1** provides a summary of the variables from each study that were used in the current analyses.

**Table 1:** Ages assessed and measures available for analysis from each study.

Study	Age (in months)	N	Social Und: EWCL+UCLA	Temp: ECBQ (fear)	Temp: ECBQ (shy)	Temp: TBAQ (social fear)	Parent socialization: PBQ	Instrum helping	Emp helping	Sharing	Parent report – child Prosocial: PBQ + SDQ
1	18, 30	66	X				X	X	X	X	X
2	18, 30	58	X				X	X	X		X
3	30	45	X	X	X			X	X		
4	18, 24	82	X			X	X			X	X
5	18, 30	74		X	X		X	X	X		

## Studies 1 and 2

Studies 1 and 2 (Waugh et al., 2013) included 71 18-months-old (*M* = 18.61, SD = 0.843), and 53 30-months-old (*M* = 28.99, SD = 0.973). Both studies included an instrumental helping task and an empathic helping task; Study 1 additionally included two sharing tasks.

In the instrumental helping task (adapted from Over and Carpenter, 2009), while E kneeled to place some things on a small table, she “accidentally” dropped six sticks on the floor on the far side of the table. During the empathic helping task (adapted from Svetlova et al., 2010) E became cold while sitting on one side of the room, having placed her blanket on a table across the room; prior to the empathic helping task, E modeled being cold and demonstrated that her blanket made her warm.

The two sharing tasks (adapted from Brownell et al., 2013a) were administered by E while AE and the child sat next to each other at side-by-side tables. AE served as a playmate and did not direct the child or the activities. The two tasks were identical but for different toys (cars; animals). E first evenly distributed several toys to both AE and the child; after a 60 s free-play period, E removed the toys from AE and the child, placed all of toys in front of the child, and moved to a corner of the room behind the child.

## Study 3

Study 3 (Drummond et al., 2014a) included 45 30-months-old (*M* = 28.73, SD = 1.136) who were administered two instrumental and two empathic helping tasks. Two of these were identical to the tasks in Studies 1 and 2 (dropped sticks; cold) and two were unique to this study. In the new instrumental helping task, E “accidentally” dropped a stack of papers from a high cabinet onto the floor while reaching into the cabinet. In the new empathic helping task, E became sad after receiving a phone call; he had previously shown the child that his favorite toy made him happy, but it was now on a table out of his reach.

## Study 4

Study 4 (Brownell et al., 2013a) included 26 18-months-old (*M* = 18.0 months; SD = 0.5) and 56 24-months-old (*M* = 23.4 months; SD = 1.45). Six sharing tasks, differing only in the toys to be shared, were administered following the same procedures as in Study 1. See Brownell et al. (2013a) for detailed description of tasks and procedures.

## Study 5

Study 5 (Svetlova et al., 2010) included 38 18-months-old (*M* = 18.46 months; SD = 0.48) and 36 30-months-old (*M* = 30.32 months; SD = 0.68). Three instrumental helping tasks and three empathic helping tasks were administered following the same procedures as in Study 3. The three instrumental helping tasks were unique to this study; two of the empathic helping tasks were identical to those used in Study 3 (cold; sad) and one was unique to this study. In the first instrumental helping task, E dropped a clothespin out of reach while clipping cloths to a clothesline (adapted from Warneken and Tomasello, 2006). In the second instrumental helping task, E ran out of wrappers while wrapping toys and the additional wrappers were out of her reach. In the third instrumental helping task, E needed a toy she had been playing with but it was out of her reach. In the new empathic helping task, E became frustrated with her messy hair hanging in her eyes and needed a hairclip that was out of her reach (E had previously demonstrated that the hairclip was used to clip her hair up and that it alleviated the frustration with her messy hair). See Svetlova et al. (2010) for detailed descriptions of tasks and procedures.

## Questionnaires

During each study, parents filled out questionnaires about their child including temperament, social understanding (two questionnaires), prosocial behavior (two questionnaires), and parental socialization practices. Cronbach’s alphas ranged from 0.79 to 0.97 across the studies. While not all studies contained the same questionnaires, the questionnaires that were used across studies were identical. Please see **Table 1** for a summary of the measures used in each study that are included in analyses.

### Social Understanding

Social understanding was measured using a composite score from the UCLA Self-Understanding questionnaire (Stipek et al., 1990) and the Emotion Words Checklist (EWCL; Brownell et al., 2006). The UCLA Self-Understanding questionnaire consists of 24 items rated on a 3-point scale (0 = definitely not; 1 = sometimes; 2 = definitely) that evaluate self-recognition, self-description, and self-evaluation in toddlers and young preschoolers. The EWCL consists of a list of 29 emotion-related words adapted from Bretherton and Beeghly (1982) and Shatz et al. (1983). Parents indicated how often the child had said each emotion word in the past 6 months (0 = Never; 1 = once or twice; 2 = three-five times; 3 = Often). Scores on each instrument were standardized and then summed to create a composite social understanding variable. Raw scores (summed) ranged from 0 to 135 (*M* = 47.513, SD = 27.985).

### Temperament

Parents rated their children’s temperament using the Early Childhood Behavior Questionnaire (ECBQ; Putnam et al., 2006) or the Toddler Behavior Assessment Questionnaire (TBAQ; Goldsmith, 1996). Of interest for the current study were the subscales of fearfulness and shyness from the ECBQ and the social fear subscale from the TBAQ. On the ECBQ parents rated 11 fear-related behaviors and 12 shyness-related behaviors on a 7-point scale according to how often parents had observed them during the previous 2 weeks (1 = Never; 2 = Very rarely; 3 = Less than half the time; 4 = About half the time; 5 = More than half the time; 6 = Almost always; 7 = Always). Children received an average fearfulness score of 1–7 (*M* = 2.545, SD = 0.87) and an average shyness score of 1–7 (*M* = 3.52, SD = 0.99). We standardized and summed the fearfulness and shyness scores to create a composite shy-fearful score. On the TBAQ parents used the same 7-point scale to rate 11 social fear-related behaviors in their children. Children received an average social fear score of 1–7 (*M* = 3.59, SD = 1.01) which was standardized for analyses.

### Parent-reported Prosocial Behavior and Socialization Practices

Parents completed the prosocial behavior subscale of the Goodman Strengths and Difficulties questionnaire (SDQ; Goodman, 1997), which contains five items (e.g., “kind to younger children;” “considerate of other people’s feelings”) rated on a 3-point scale (0 = Not True; 1 = Sometimes True; 2 = Certainly True); children received a total score of 0–10 (*M* = 6.24, SD = 1.87). Parents also completed a questionnaire (Prosocial Behavior Questionnaire; PBQ) developed to assess their socialization practices related to prosocial behavior (e.g., “Ask my child to help even if I don’t really need it, just for the purpose of teaching him/her about helping;” “Praise/thank my child when s/he helps me or someone else;” “Talk about my child’s and other people’s feelings with my child”) as well as the child’s demonstration of prosocial behavior at home (e.g., “Tries to help me around the house;” “Willingly shares food or toys with a parent without being asked”). Parents rated 12 socialization items and 14 child prosocial behavior items on a 5-point Likert scale (0 = Not at all; 1 = Once or twice; 2 = Sometimes, a few times a month; 3 = Often, a few times a week; 4 = All the time, everyday), yielding total scores ranging from 0–48 (*M* = 36.83, SD = 8.00) and 0–56 (*M* = 36.18, SD = 7.80), respectively. Scores for children’s prosocial behavior from the SDQ and from the PBQ child prosocial behavior subscale were standardized and summed to produce a total parent-reported prosocial behavior variable.

## Results

The primary goal of the current study was to examine relations between individual differences in toddlers’ social understanding and prosocial behavior, as well as parent socialization and several dimensions of temperament. To examine predictors of parent-reported prosocial behavior, we calculated partial correlations, controlling for age and gender (where appropriate), among social understanding, parent socialization, temperament, and parent-reported prosocial behavior. To examine predictors of the observed measures of prosocial behavior from the lab tasks, we calculated one-way ANCOVAs, controlling for age and gender (where appropriate), with the categorical helping/sharing variable as the independent variable, and temperament, social understanding, and parent socialization as dependent variables. **Table 1** specifies which studies contributed which variables for analyses, and **Table 2** provides descriptive information for the raw (unstandardized) scores.

**Table 2:** Descriptive statistics (mean and SD) for raw (unstandardized) data.

Study	Age (in months)	*N*	Social und	Temp (ECBQ): fear	Temp (ECBQ): shy	Temp (TBAQ): social fear	Parent socialization	Lab: instrum helping^a^	Lab: emp helping^a^	Lab: sharing^a^	Parent report: child prosocial^b^
1	18	33	24.78 (16.26)				35.88 (7.85)	20 (61%)	12 (38%)	28 (85%)	3.95 (0.87)
	30	33	71.42 (17.70)				38.42 (5.17)	30 (91%)	26 (79%)	30 (91%)	4.24 (0.62)
2	18	38	22.59 (9.58)				35.54 (5.26)	23 (61%)	17 (45%)		3.92 (0.94)
	30	20	78.65 (17.61)				40.57 (2.82)	18 (95%)	15 (75%)		4.14 (0.72)
3	30	45	71.10 (23.70)	2.45 (0.87)				44 (98%)	43 (96%)		
4	18	26	23.24 (10.00)			30.20 (11.31)	36.72 (6.77)			25 (96%)	3.18 (0.60)
	24	56	46.11 (20.61)			34.55 (12.10)	37.42 (6.60)			56 (100%)	3.52 (0.75)
5	18	38		2.53 (0.92)	3.58 (1.04)		33.71 (6.76)	32 (100%)	31 (97%)		
	30	36		2.67 (0.83)	3.46 (0.98)		38.19 (6.29)	33 (100%)	33 (100%)		

*^a^Scores are dichotomous (helped/shared at least once vs. never helped); cell entries are counts (percentages).*

*^b^Scores are means (SD) of the sum of the average scores on the two parent-report measures, the ESB and PBQ (range = 0 – 6).*

### Preliminary Analyses

Preliminary analyses revealed, as would be expected, that age was positively correlated with social understanding [*r*(229) = 0.78, *p* < 0.001], parent socialization practices [*r*(236) = 0.24, *p* < 0.001], and parent-reported prosocial behavior [*r*(193) = 0.19, *p* < 0.01]. Furthermore, children who helped were significantly older than those who did not, for both instrumental helping [*M* = 25.15 mos. vs. *M* = 19.89 mos.; *F*(1,231) = 30.017, *p* < 0.001] and empathic helping [*M* = 25.53 mos. vs. *M* = 21.06 mos.; *F*(1,231) = 32.841, *p* < 0.001]. Because the focus of this paper is on individual differences rather than age differences, age is controlled in all analyses.

Analyses revealed few differences by gender. Girls were rated significantly higher than boys on temperamental fear [all scores standardized; *M* = 0.33 vs. *M* = -0.34; *F*(1,110) = 12.640, *p* < 0.001], as well as on the shy-fearful composite score [*M* = 0.36 vs. *M* = -0.32; *F*(1,66) = 8.685, *p* < 0.01]; girls and boys did not differ on the social fear subscale. Girls had significantly higher social understanding scores [*M* = 0.22 vs. *M* = -0.18; *F*(1,227) = 9.515, *p* < 0.01]. Girls also had significantly higher scores on parent-reported prosocial behavior [*M* = 0.33 vs. *M* = -0.26; *F*(1,191) = 5.75, *p* < 0.05] and were marginally more likely to help in empathic helping situations (85 vs. 71%; χ^2^ = 3.693, *p* < 0.1, *n* = 233). Because gender differences were not systematic, gender is controlled only when relevant.

Finally, to examine whether different measures of prosocial behavior were associated, we conducted a series of chi-square analyses between each of the observed categorical helping/sharing scores, and point bi-serial correlations between parent-reported prosocial behavior and the observed categorical helping/sharing scores. Results showed that empathic helping was significantly related to instrumental helping (χ^2^ = 55.98, *p* < 0.001, *n* = 232), sharing (χ^2^ = 7.94, *p* < 0.01, *n* = 65), and parent-reported prosocial behavior (*r*_pb_ = 0.19, *p* < 0.05, *n* = 122). Instrumental helping was significantly related to parent-reported prosocial behavior (*r*_pb_ = 0.21, *p* < 0.05, *n* = 122), but not sharing (χ^2^ = 0.003, *ns*, *n* = 66). Sharing and parent-reported prosocial behavior were unrelated (*r*_pb_ = -0.15, *ns*, *n* = 144. This variability in associations among various types of prosocial behavior is consistent with other recent findings with toddlers (e.g., Dunfield et al., 2011) and with larger conceptualizations of prosocial behavior as a multifaceted, multidimensional construct, with distinct subtypes that call on unique as well as overlapping skills, understanding, and motivations (Thompson and Newton, 2013; Dunfield, 2014; Eisenberg and Spinrad, 2014).

### Associations between Social Understanding and Prosocial Behavior

The first aim was to determine associations between individual differences in social understanding and individual differences in prosocial behavior. For parent-reported prosocial behavior, the partial correlation controlling for age and gender yielded a significant association with parent-reported social understanding [*r*(176) = 0.18, *p* < 0.05]. A series of one-way ANCOVAs controlling for age was then conducted with each of the observed categorical helping/sharing variables as factors and parent-reported social understanding as the outcome. Children who helped in at least one empathic helping task, compared to those who did not help in any, had significantly higher social understanding scores [*M* = 0.28 vs. *M* = -0.56; *F*(1,77) = 5.503, *p* < 0.05]. There were no differences in social understanding for children who helped instrumentally vs. those who did not, or for children who shared vs. those who did not. Thus, early individual differences in social understanding are associated with both parent-reported prosocial behavior and observed empathic helping, but not with sharing or instrumental helping.

### Does Parent Socialization or Temperament Predict Social Understanding?

The second aim was to determine whether individual differences in parent socialization or temperament were associated with differences in social understanding. Parent socialization was significantly positively correlated with toddlers’ social understanding, controlling for age and gender [*r*(156) = 0.28, *p* < 0.001]. However, no significant associations were found between any of the temperament measures and social understanding, controlling for age and gender.

### Does Parent Socialization or Temperament Predict Prosocial Behavior?

The third aim was to determine whether individual differences in parent socialization or in temperament were associated with individual differences in prosocial behavior. For parent-reported prosocial behavior, partial correlations controlling for age yielded significant associations with parent socialization [*r*(158) = 0.42, *p* < 0.001], but not with temperament. A series of one-way ANCOVAs controlling for age was then conducted with each of the observed categorical helping/sharing variables as factors and parent socialization and temperament as outcomes. Results revealed that parent socialization practices did not differ between children who helped or shared at least once on and those who did not; nor were there were differences in shy/fearful temperament between those children who helped or shared and those who did not. Thus, similar to the findings for social understanding, variability in parent socialization with their toddlers was associated with variability in parent-reported prosocial behavior.

## Discussion

With the growing number of demonstrations of the remarkable prosociality of children in their second year of life, attention has recently focused on identifying age-related changes and potential developmental mechanisms underlying this capacity (e.g., Warneken and Tomasello, 2009; Brownell, 2013; Barragan and Dweck, 2014; Carpendale et al., 2014; Dunfield, 2014; Paulus, 2014). Much less attention has been paid to the question of individual differences: why are some young children more likely to behave prosocially than others? One obvious possibility lies in individual differences in empathic concern for others’ distress, known from several decades of study to differ among children when it first makes its appearance in the second year (e.g., Bischof-Köhler, 1991; Zahn-Waxler et al., 1992; Roth-Hanania et al., 2011). In the current study, we have looked to another potential source, individual differences in early social understanding.

Because early developments in prosocial behavior and social understanding are universal normative accomplishments, we have conceptualized the sources of individual variability in these capacities as arising from either dispositional differences or differences in rates of development. That is, even as early as the second year of life, some children may be dispositionally or temperamentally more interested in, attentive to, and reflective about others’ emotions, needs, and desires than other children. Or some children may be more developmentally advanced in such abilities than others, which can occur for many reasons; here we focused on parental socialization as a potential contributor. There is evidence that differences in both disposition and rate of development relate to social understanding and prosocial behavior in childhood, as previously reviewed, but little evidence for when in development such associations arise.

In the current report, combining data from several previous studies of helping and sharing in 1- and 2-years-old, we found that individual variability in social understanding predicted both parent-reported prosocial behavior at home and whether children were likely to help an adult with emotion-based, empathic helping tasks in the lab. Social understanding did not predict children’s instrumental helping in the lab. Examining potential sources of variability in both social understanding and prosocial behavior, we found no evidence that variability in either was associated with the temperamental quality of a fearful, shy or socially cautious stance, as has previously been shown in childhood (e.g., Wellman et al., 2011; Mink et al., 2014). In contrast, we did find that parental socialization of behavior linked with prosocial responding, which is likely to affect rates of development, was associated with individual differences in both social understanding and prosocial behavior. We discuss these findings in greater detail below.

### Individual Differences in Social Understanding Predict Prosocial Behavior

Children’s social understanding in the current study was assessed by parent report of their self-understanding (e.g., mirror self-recognition; pride) and their understanding and use of emotion and internal state words (e.g., happy, sad, hungry). Controlling for age differences in both social understanding and prosocial behavior, 18- to 30-months-old children with more advanced levels of social understanding were also more likely to help an adult who was cold or sad by bringing a blanket or favorite toy to alleviate the adult’s distress, and were reported by their parents to demonstrate prosocial behavior more often in daily life.

These findings are consistent with larger conceptual frameworks in which early-emerging social understanding is hypothesized to contribute to the genesis of prosocial responding in the second year of life (e.g., Vaish et al., 2009; Brownell et al., 2013b; Paulus, 2014). They are also consistent with the handful of other studies that have directly assessed some aspect of social understanding in toddlers (e.g., fairness; joint attention; personal pronouns; intention understanding) and concurrently assessed some form of prosocial behavior (e.g., sharing, helping, cooperation) and have reported positive associations (e.g., Ensor and Hughes, 2005; Brownell et al., 2006; Nichols et al., 2009; Sommerville et al., 2013; Kartner et al., 2014; Newton et al., 2014).

Unique to this study, we extended the association with prosocial behavior in previous laboratory-based studies to toddlers’ everyday prosocial behavior in the family. This is important for demonstrating that the particular opportunities or challenges characteristic of laboratory tasks are not what drives these early-appearing associations between social understanding and prosocial behavior. Rather, variability in social understanding relates to nascent prosocial behavior in the everyday life of the home as well. As previous scholars have argued, family contexts are not only the primary setting in which prosocial behavior arises, but are also distinctively demanding of social understanding (e.g., Dunn and Munn, 1985; Thompson and Newton, 2013).

We also found that variability in social understanding differentially predicted empathic versus instrumental helping in the lab. Notably, in our tasks, the superficial task demands are identical across instrumental and empathic helping: the child needs to bring the adult an object that the child, but not the adult, can reach which will permit the adult to achieve a goal (instrumental helping) or will alleviate the adult’s negative internal state such as being cold or sad (empathic helping). What differs between the two types of scenarios is the nature of the social understanding required. For instrumental helping, the problem is immediate, concrete, and requires recognition of another’s goals, an ability that infants are known to be capable of in the first year of life (Woodward, 2005). For empathic helping, however, the inferences are more complex and abstract, requiring some understanding of the links among facial and bodily expressions, subjective states, the contextual factors that give rise to particular internal states, and how particular actions can alter another’s internal state. It is telling, therefore, that a general measure of social and emotional understanding as reported by parents predicted just the sort of prosocial responding that depends on more complex inferences about others’ internal states. This finding also adds to the growing body of evidence that instrumental and empathic helping may derive from different underlying mechanisms (Paulus et al., 2013; Dunfield, 2014).

### Does Temperament Predict Individual Variability in Social Understanding or Prosocial Behavior?

One potential source of within-age variability in social understanding and prosocial behavior is dispositional differences in children’s interest in others’ emotions and mental states or their motivation to intervene in them. However, in contrast to the intriguing, albeit still limited evidence for associations between a shy, socially fearful temperament and advanced social reasoning in preschool children (Wellman et al., 2011; Mink et al., 2014), in the current study with toddlers we found little evidence for such temperament differences predicting concurrent social understanding. Previous studies have reported modest, but significant, associations with shyness or social fear rather than with more generalized fear such as fear of the dark or of loud noises. In the current data, no relations were found with either type of fearfulness. This may be because the mechanism presumed to underlie the association with ToM in older children, a more reflective stance on others’ behavior and mental states (e.g., Wellman et al., 2011), does not yet hold for toddlers. Or the association found in prior studies may be unique to more advanced forms of social reasoning such as those underlying false-belief understanding. Finally, it is also possible that measurement differences could account for the lack of effects in the current study as our measure of social understanding differed not only in substance from the theory-of-mind measures used in previous studies, but it was also parent-reported rather than assessed directly with the child. Additional research will be needed to sort out these possibilities and to determine when in development dispositional differences begin to predict variability in social understanding.

As for prosocial behavior, previous work has been mixed as to the nature of the links between these dimensions of temperament and toddlers’ prosocial responses. In line with prior failures to find associations between temperamental fearfulness and prosocial helping and comforting in 1- and 2-years-old (Spinrad and Stifter, 2006; Liew et al., 2011), we found no associations between either social fear or a composite measure of shyness–fearfulness and either parent-reported prosocial behavior or observed helping or sharing in toddlers. Worth noting is that the temperament measures in the current study and one of the measures of prosocial behavior were parent-reported, yet even with the potential for shared method variance, no associations were found. Given that at least three independent studies using a variety of measures for these temperament constructs and for prosocial behavior have failed to find significant associations between them, one might be tempted to conclude that such dispositional variability in very young children is not an important factor in predicting individual differences in emerging prosocial behavior. However, this very variety in measurement may itself limit the conclusions that can be drawn, and points to the need for more systematic and focused research on the question. Additionally, we examined only three dimensions of temperament. It is quite possible that other temperament dimensions (e.g., effortful control; sociability) may relate more strongly to social understanding or prosocial behavior in this age group.

### Socialization of Social Understanding and Prosocial Behavior

A second potential source of within-age variability in social understanding and prosocial behavior is parents’ efforts to socialize young children’s attention to and recognition of others’ emotions, needs, and desires and their children’s caring for and prosocial action on behalf of others. Here we did find associations between toddlers’ social understanding and how often parents reported that they engaged in practices such as asking their toddlers to help them, using facial expressions or gestures when requesting help, and talking about the child’s or others’ feelings. A growing body of research has found other similar links between socialization and social understanding among toddler-aged children, with some of the studies longitudinal and the effects putatively causal (e.g., Dunn and Munn, 1985; Dunn et al., 1991; Symons et al., 2006; Taumoepeau and Ruffman, 2006; Ensor and Hughes, 2008; Brownell et al., 2013c). Many of these have examined the role of parental talk about emotions and mental states, which is likely to promote young children’s perspective taking and consideration of others’ needs. Although our measure of parent socialization also included items that refer to parents’ talk about feelings, most of the items refer to parents’ encouragement of prosocial behavior. Interestingly, then, our findings suggest the possibility that, for very young children at least, engagement in prosocial actions within the family may be another route through which perspective taking and appreciation of others’ needs and desires could arise.

We also found associations between parents’ socialization of prosocial behavior and their report of children’s prosocial behavior in the family. This suggests that parents’ routine encouragement of their toddlers’ participation in prosocial action in the context of everyday household routines and activities promotes young children’s everyday prosocial responding. Recent observations of parents and toddlers at home (Dahl, 2015) have shown that 11- to 25-months-old children are, indeed, frequently encouraged and supported by other family members in everyday helping, and that toddlers participate in a wide range of activities that are especially geared toward assisting others with immediate goals (e.g., cleaning up; sweeping; handing an object that someone else needs). By including the child in routine activities with shared goals, parents may help children figure out how to assist others with their goals and may communicate more general norms and expectations about helpfulness, especially when this occurs in the context of reciprocal, responsive interactions (Ensor and Hughes, 2008; Barragan and Dweck, 2014).

## Conclusion

From the current results are subject to several limitations. First, the use of parent-report measures has both advantages and disadvantages. On the one hand, parents observe their children over time and multiple contexts, providing a broader and possibly more valid assessment of their children’s competence than lab observations; they are also in the best position to evaluate their own socialization goals and practices. On the other hand, questions about shared method variance and reporter bias, including social desirability, are inevitable. However, we have used multiple measures of prosocial behavior in the current study, including observed behavior, offsetting some of these concerns; and much of the research on parenting, social understanding, and prosocial behavior conducted in childhood uses parent-report (see Eisenberg et al., 2015, for a review) which provides further confidence in the current findings. Second, the cross-sectional and correlational design precludes causal claims. For example, the association between parent socialization and social understanding could be due to the possibility that parents who are more enthusiastic about encouraging their children to help and share are also more likely to engage in emotion and mental-state talk with their children or to be more sensitive to evidence of early social understanding in their child’s behavior. Furthermore, it is worth noting that other factors besides those on which we’ve focused in the current study undoubtedly contribute to individual differences in social understanding and prosocial behavior. These include child factors such as motivational, attentional, and regulatory capacities; parent characteristics, such as warmth and reciprocity, and aspects of the parent–child relationship, such as attachment security. Moreover, these are likely to interact with one another and with the constructs examined in this paper to influence early individual differences.

In sum, the current study has shown that individual variability in early social understanding is associated with variability in several different measures of prosocial behavior, and that variability in both constructs is more strongly and consistently associated with differences in parents’ socialization of prosocial responding than with dispositional differences among children.

## Conflict of Interest Statement

The authors declare that the research was conducted in the absence of any commercial or financial relationships that could be construed as a potential conflict of interest.
